# Genomic medicine and the “loss of chance” medical malpractice doctrine

**DOI:** 10.1016/j.xhgg.2021.100032

**Published:** 2021-04-05

**Authors:** Jennifer K. Wagner, Michelle N. Meyer

**Affiliations:** 1 Center for Translational Bioethics and Health Care Policy, Geisinger, 100 N. Academy Ave., MC 30-42, Danville, PA 17822, USA; 2 Steele Institute for Health Innovation, Geisinger, 100 N. Academy Ave, Danville, PA 17822, USA

**Keywords:** ELSI, genetics, precision medicine, liability, professional responsibility

## Abstract

As genomic medicine expands, interest in how medical malpractice law will apply to such questions as whether and when to return new or updated genomic results has grown. Given that access to some genomic results (such as those pertaining to minors or those for which scientific interpretations are unsettled) is delayed for years, the “loss of chance” (LOC) doctrine is of particular potential relevance. Yet it has received relatively little attention among scholars of law and genomics. We performed legal research to determine the status of this malpractice doctrine across the United States and consider its potential applicability to genomic medicine. We further examined known genomic medicine malpractices to assess whether this doctrine had yet been invoked in that context. We identified a trend toward adoption of the LOC doctrine, finding 29 states (58%) have adopted, 15 states (30%) have rejected, and six states (12%) have deferred or not yet addressed the doctrine. Attempts to invoke or apply the doctrine in the known genomic medical malpractice cases were also found. While our findings do not provide cause for substantial concern, the availability of the LOC medical malpractice doctrine is a potentially important factor to consider when making programmatic decisions for genomic medicine. Future research examining whether liability risks posed by this doctrine prompt defensive medicine practices would be useful.

## Teaser

The “loss of chance” medical malpractice doctrine is available in a majority of jurisdictions in the United States and should be considered when making programmatic decisions for genomic medicine. It has particular significance regarding programmatic decisions to delay or withhold access to genomic results from the individuals to whom those results pertain.

## Introduction

Genomic medicine malpractice caselaw is only beginning to emerge, with approximately 200 reported cases over four decades that involve alleged failures to diagnose a genetic disorder, interpret genetic test results appropriately, offer genetic screening when indicated, return results to patients, or treat a genetic condition properly.^1^^,^^2^ Nevertheless, genetic professionals have long been concerned about possible legal liability when performing professional tasks, such as those involving the scope of testing, access to variant data and/or interpreted results, and recontact to provide relevant updates as genomic science and technology advance.^3^, ^4^, ^5^, ^6^, ^7^, ^8^, ^9^ These include liability risks that might attach when decisions are made to return or, alternatively, withhold data or results from individuals to whom those pertain.

Although the law is not clear, most^10^, ^11^, ^12^—but not all^13^—commentators have distinguished between medical professionals’ responsibilities to research participants and their responsibilities to patients, arguing that the former do not rise to the level of the latter. This position arguably reflects a broader ethical and regulatory distinction between research and practice.^14^ In the specific context of genomic medicine and research, commentators have similarly distinguished between obligations to patients and obligations to research participants.^15^, ^16^, ^17^, ^18^, ^19^ However, when broad genomic research is conducted in contexts such as learning health systems,^20^ in which participants are also patients and research is deliberately embedded in the routine practice of medicine, application of the so-called “research-practice distinction” is unclear, even assuming that medical professionals’ legal duties to individuals track such a distinction in the first place.

When institutional policy or a research protocol stipulates that data or results will be withheld (regardless of underlying rationale), when an erroneous test result is returned, or when a test result is returned that reflects the best known evidence at the time but is later upended by new data with no corresponding update to the patient—especially when the nature of the action as research or practice is ambiguous—there are legitimate concerns about when and how individuals might ultimately learn this information and whether the discovery will be too late for those individuals to avoid (1) the progression of a condition for which prevention or treatment was available, or (2) unnecessary harms, such as ineffective treatments for which substitutes were available.

Consider the following real-world examples:

1. Pediatric lipid screening. Those with familial hypercholesterolemia (FH) (MIM:143890) have a 20- to 100-fold greater risk of cardiac events before the age of 50 years, compared to the general population. Evidence suggests that the benefits for preventing cardiovascular disease of lipid-lowering medications (LLMs) are greater when they are administered throughout the life course than when they are not initiated until middle age. Until relatively recently, a pediatric guideline recommended selective screening for genetic dyslipidemias, including FH, based on family or personal history. In 2011, motivated by the benefits of lifelong use of LLMs and likely deficiencies of targeted screening, the American Academy of Pediatrics and the National Heart, Lung, and Blood Institute recommended universal lipid screening for children between the ages of 9 and 11 years and for young adults between the ages of 17 and 21 years. The guideline is intended to benefit not only children themselves but also their parents, who might be identified as having FH through reverse cascade testing (i.e., systematic, routine screening of first-degree relatives following a genetic result for a child). Despite this guideline, a study of five health systems found that rates of pediatric lipid testing remain low and, in fact, have decreased between 2002 and 2012 from an estimated 16% to 11%.^21^
2. Research biobanks that return clinically actionable results. Geisinger’s MyCode Community Health Initiative (MyCode) is a research biobank that enrolls any and all consenting Geisinger patients, sequences their whole exomes, and conducts basic research on, e.g., variants of unknown significance. However, in addition, the program deliberately searches participants’ research exome sequences for clinically actionable pathogenic or likely pathogenic variants. It confirms these “research results” in a Clinical Laboratory Improvement Amendments (CLIA)-certified laboratory, reports them to both the patient-participant and their primary care physician, and deposits them in the electronic health record (EHR) for ongoing risk management according to clinical best practice guidelines. If either a research or a CLIA result is not, at the time of analysis, considered to be pathogenic or likely pathogenic, it is not returned to the patient-participant or her provider, nor is it noted in the EHR. Participants are told that “no news means no news.”^22^
3. Clinical whole-exome sequencing. More recently, Geisinger began offering CLIA-grade whole-exome sequencing to patients outside of the MyCode protocol as a clinical service. This clinical test screens for the same variants that are returned to MyCode research participants. Unlike MyCode, all results, negative or positive, are returned to patients and their providers via the EHR.^23^

A successful genomic medical malpractice claim requires the plaintiff to prove all elements of the cause of action: a duty existed, the defendant breached that duty, and the breach caused damages. Under traditional medical malpractice law, a plaintiff cannot satisfy the last of these elements unless she can show by a preponderance of the evidence that the defendant’s negligence more likely than not caused her injury. Nondisclosed, erroneous, or delayed genetic testing can, in turn, delay treatment that might reduce the risk of the underlying genetic condition. But is such negligence the proximate cause of injury—say, a cardiac event? To draw on one of the examples above, suppose that a doctor fails to adhere to current guidelines and does not order a lipid panel for a 10-year-old, missing the opportunity to detect a pathogenic FH variant. The FH is diagnosed, and LLMs begun, only years later, when they are less helpful in preventing cardiac events. Although the underlying FH variant is a but-for cause of the injury, if the patient can show that similarly situated patients with more timely accurate genetic testing have, say, a 70% chance of avoiding such an event, whereas with delayed testing and uptake of risk management measures have only a 10% chance, then providing expert evidence of this will probably allow her to recover. But if her chance of avoiding a cardiac event were less than *50%* even with timely, accurate, and disclosed genetic testing, traditional tort law would deny her recovery, since she would not be able to show that the proximate cause of her injury is the medical negligence.

The “loss of chance” doctrine (LOC doctrine) is a rule available in some US states that allows plaintiffs to prove that a breach caused damages, when without the doctrine they otherwise would not be able to satisfy that element. Specifically, the LOC doctrine enables claims for medical malpractice when the doctor’s negligent actions have reduced or eliminated opportunities for better outcomes (either by decreasing the chance of recovery or survival from pre-existing conditions or by increasing the risk of harm).^24^ The factual circumstances in which application of the LOC doctrine is sought could involve delayed or erroneous diagnoses as well as delayed or erroneous treatments, and they could allege physical health, mental health, or non-health harms. The LOC doctrine first appeared in a 1966 US court opinion involving an alleged loss of chance to survive a bowel obstruction due to a negligent failure to properly diagnose it. The doctrine did not appear as the subject of a scholarly article until 15 years later.^25^^,^^26^ Additionally, the American Law Institute, which issues Restatements “to clarify, modernize, and otherwise improve the law,”^27^ has declined to take an official position on the LOC doctrine.^28^ Despite the acknowledgment by some Ethical, Legal, and Social Implications (ELSI) research scholars of the LOC doctrine’s relevance to the issuance of secondary genomic findings,^29^^,^^30^ there is a paucity of critical analysis of the LOC doctrine as it relates to genomic medicine and research. More fundamentally, the most recent, otherwise comprehensive empirical study of genomic medical malpractice cases does not report whether the LOC doctrine was invoked in any of the relevant cases.^1^ In short, it is not known whether the doctrine has ever been invoked in a medical malpractice case concerning genomics.

In a jurisdiction where the LOC doctrine has either been rejected or deferred, the traditional medical malpractice burdens of proof would apply and often prevent patients from recovering damages. Conversely, the LOC doctrine is friendlier to patients with pre-existing conditions, and, in a jurisdiction where the LOC doctrine has been adopted, patients could pursue remedies for the lost chance even if the patient’s ultimate prognosis regardless of the negligence is grim. To revisit the FH example used above to illustrate, in a jurisdiction where the LOC doctrine has been adopted—but only in such a jurisdiction—an individual could successfully seek compensation for the lost chance attributable to the delay, even if the ability to avoid a cardiac event with proper care was only 50%.

The status of the LOC doctrine throughout the United States could have important implications for consistency in experiences for patient-participants and genetic professionals in geographically dispersed precision health initiatives. For example, Geisinger’s MyCode enrolls patient-participants in multiple states (specifically, Pennsylvania and New Jersey) and has no restrictions requiring patient-participants to reside in those two states in order to be eligible.^31^ As a result, state variation in the LOC doctrine could mean that programmatic decisions (e.g., regarding precisely what genetic information is CLIA-confirmed and what, when, and how genomic information is ultimately used in genomic medicine practice) raise varying liability risks for the genetic professionals involved.^15^^,^^32^ Clarifying the state variability of particular legal issues is also increasingly recognized as important for the design of trustworthy technology to enable interoperability of health records and the design of universal informed consent approaches.^33^^,^^34^

In light of this, we sought to better understand the status of the LOC doctrine in the context of the practice of genomic medicine and research.

## Material and methods

We used WestlawNext (Thomson Reuters) to conduct a survey of the LOC doctrine in each of the 50 US states to determine whether the doctrine is viable in any medical malpractice context (not merely the narrow context of genomic medicine). Relying upon the legal principle of *stare decisis*, defined as “[t]he doctrine of precedent, under which a court must follow earlier judicial decisions when the same points arise again in litigation,”^35^ we examined the precedential value of the citations and characterizations provided by earlier legal scholars who in 2015 examined the LOC doctrine broadly across all areas of law (including, e.g., the doctrine applied to general medical malpractice cases and variants of the doctrine applied to legal malpractice cases and cases involving other areas of law).^36^ Citations involving negative treatment from controlling authority were reviewed to determine whether the negative treatment involved the court’s recognition of the LOC doctrine or merely other portions of the case not affecting its precedential value. To be comprehensive, we also examined the status of the LOC doctrine in the District of Columbia and five US territories (i.e., Puerto Rico, Virgin Islands, American Samoa, Guam, and Northern Mariana Islands) by designating that jurisdiction, searching the term “loss of chance doctrine,” and reviewing the resulting citations identified (if any). Determinations of the status of the LOC doctrine in each of the 56 jurisdictions (i.e., adopted, rejected, deferred, or not yet addressed) were reached by consensus. Figures illustrating the variation of results across the jurisdictions through customized maps were created using mapchart.net.

Additionally, the genomic malpractice cases identified in a previously published empirical study of genomic malpractice cases decided through December 31, 2016 (N = 202) without regard to the LOC doctrine were revisited with the LOC doctrine specifically in mind to consider (1) whether those cases were decided in jurisdictions determined by us to have adopted the LOC doctrine, (2) whether those cases involved any adult-onset conditions (as withholding or delaying return of a genetic result for such a condition to a minor patient-participant might implicate the LOC doctrine’s applicability), and (3) whether the LOC doctrine was specifically invoked in those cases as discernable from the court opinions or jury verdict and settlement summaries. There are two notable limitations to our approach with this secondary analysis. One limitation is that the date upon which the case was decided relative to the date upon which the LOC doctrine was adopted or rejected in that jurisdiction was not assessed; thus, it is not possible for us to speculate as to whether the LOC doctrine would have been a viable theory in those specific cases. A second limitation is that for some cases only the verdict and settlement summary documents were accessible for the analysis, and these summaries might not include sufficient detail about the legal arguments raised or the factual allegations to decipher whether the LOC doctrine was invoked or not. Thus, our analysis potentially underestimates the frequency with which the LOC doctrine has been invoked in genomic malpractice cases. Five cases were excluded from the final component of this secondary analysis (i.e., whether LOC doctrine had been invoked) because, even with the assistance of law librarian support, relevant source materials could not be located. These cases are: Anonymous Parents v. Anonymous Physicians, 2008 WL 6101332; Anonymous female v. Anonymous OB/Gyn, 2009 WL 6866508; Anonymous, 10/28/2013 Va. Law Wkly. (VA) 2013 WLNR 27451925 (2013); Cross v. Chien, 2013 WLNR 13465700 (Jury Verdict, St Louis County Circuit Court, 2013); and Anonymous, 2014 WLNR 37096453 (Va. 2014). The other two components (i.e., jurisdiction and condition) for these cases were discernible from the previously reported analysis.

## Results

**Figure 1:**
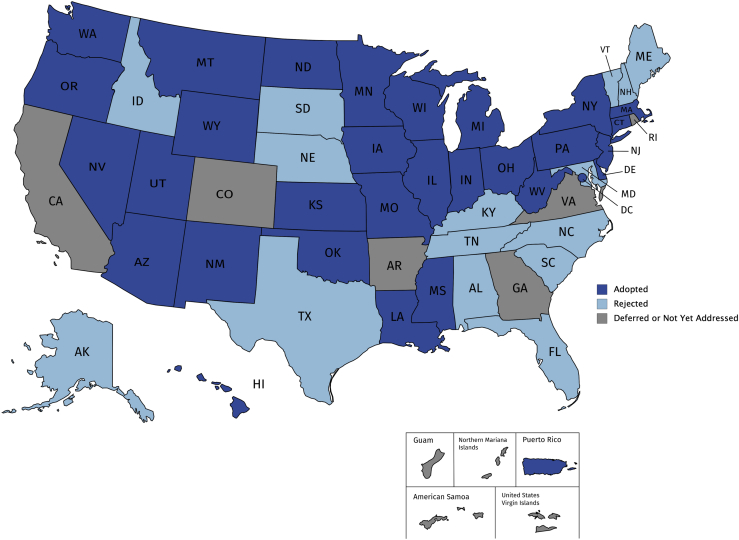
The status of the “loss of chance” (LOC) doctrine Dark blue shading indicates the LOC doctrine has been adopted. Light blue shading indicates the LOC doctrine has been rejected. Grey shading indicates the LOC doctrine has been deferred or has not yet been addressed.

Table 1 and Figure 1 display the status of the LOC doctrine in the United States as of November 11, 2019. Looking exclusively at the 50 states, a majority (29; 58%) have adopted the LOC doctrine, 15 (30%) have rejected it, and a few (6; 12%) have either deferred a decision or not yet addressed the doctrine. In recent years, there has been a modest trend toward adoption of the LOC doctrine, as shown in Table 2 by the side-by-side comparison of our findings with those published 5 years ago.^36^ A listing of the major cases contributing to the current categorization of the LOC doctrine in each jurisdiction is provided in Table S1.

**Table 1:** Status of the “loss of chance” medical malpractice doctrine in the United States

Status	No.	Jurisdictions
Adopted	31	Arizona, Connecticut, Delaware, District of Columbia, Hawaii, Illinois, Indiana, Iowa, Kansas, Louisiana, Massachusetts, Michigan, Minnesota, Mississippi, Missouri, Montana, Nevada, New Jersey, New Mexico, New York, North Dakota, Ohio, Oklahoma, Oregon, Pennsylvania, Puerto Rico, Washington, West Virginia, Wisconsin, Wyoming, Utah
Rejected	15	Alabama, Alaska, Florida, Idaho, Kentucky, Maryland, Maine, Nebraska, New Hampshire, North Carolina, South Carolina, South Dakota, Tennessee, Texas, Vermont
Deferred	4	Arkansas, Colorado, Rhode Island, Virginia
Not yet addressed	6	American Samoa, California, Georgia, Guam, Northern Mariana Islands, Virgin Islands

The status of the LOC doctrine in each of the 50 states, District of Columbia, and territories as of November 11, 2019.

**Table 2:** 50-state survey of the “loss of chance” medical malpractice doctrine

	As per Guest, Schap, and Tran^36^		As per Wagner and Meyer		
Status	No.	States	No.	States	Basis for status change
Adopted	24	Arizona, Delaware, Illinois, Indiana, Iowa, Kansas, Louisiana, Massachusetts, Minnesota, Missouri, Montana, Nevada, New Jersey, New Mexico, New York, North Dakota, Ohio, Oklahoma, Pennsylvania, Utah, Washington, West Virginia, Wisconsin, Wyoming	29	Arizona, Connecticut, Delaware, Hawaii, Illinois, Indiana, Iowa, Kansas, Louisiana, Massachusetts, Michigan, Minnesota, Mississippi, Missouri, Montana, Nevada, New Jersey, New Mexico, New York, North Dakota, Ohio, Oklahoma, Oregon, Pennsylvania, Washington, West Virginia, Wisconsin, Wyoming, Utah	Connecticut: (2008). Peterson v Ocean Radiology Associates, PC, 109 Conn. App. 275, 277–78, 951 A.2d 606. See also Superior Court of Connecticut (2014) Sawicki v New Britain General Hosp., 2014 WL 7156497.
					Hawaii: (2018). Estate of Frey v Mastroianni, 142 Hawai’i 483 (Intermediate Ct App), certiorari granted, 2018 WL 6251441 (Nov. 29, 2018)
					Michigan: See (2009) Notes of Decisions for MI ST 600.2912a and Ykimoff v Foote Mem. Hosp. 776 N.W. 2d 114, 285 Mich. App. 80, appeal denied, 791 N.W.2d 123, 488 Mich. 988, reconsideration denied, 795 NW2d 819, 489 Mich. 875
					Mississippi: (2019) Hyde v Martin, 264 So.3d 730, 732 and 734–735
					Oregon: (2017) Smith v Providence Health & Services-Oregon, 361 Or. 456, 393 P.3d 1106; see also (2018) Tomlinson v Metropolitan Pediatrics LLC, 362 Or. 431, 412 P.3d 133
Rejected	17	Alabama, Alaska, Connecticut, Florida, Idaho, Kentucky, Maryland, Michigan, Mississippi, Nebraska, New Hampshire, Oregon, South Carolina, South Dakota, Tennessee, Texas, Vermont	15	Alabama, Alaska, Florida, Idaho, Kentucky, Maryland, Maine, Nebraska, New Hampshire, North Carolina, South Carolina, South Dakota, Tennessee, Texas, Vermont	Alaska: (2017) Tate v United States, 2017 WL 902850 (D. Alaska)
					Maine: (2012) Samaan v St. Joseph Hosp., 670F.3d 21
					North Carolina: (2019) Parkes v Hermann, 828 S.E.2d 575
Deferred	4	Arkansas, Colorado, Maine, Rhode Island	4	Arkansas, Colorado, Rhode Island, Virginia	Virginia: (2014) Wagoner v Commonwealth, 63 Va. App. 229, 256
Not yet addressed	5	California, Georgia, Hawaii, North Carolina, Virginia	2	California, Georgia	

Displayed is a side-by-side comparison of the categorizations made by Guest, Schap, and Tran ^36^ with those made in this work (as of November 11, 2019). The rightmost column contains legal authority for instances of status changes or discrepancies.

**Figure 2:**
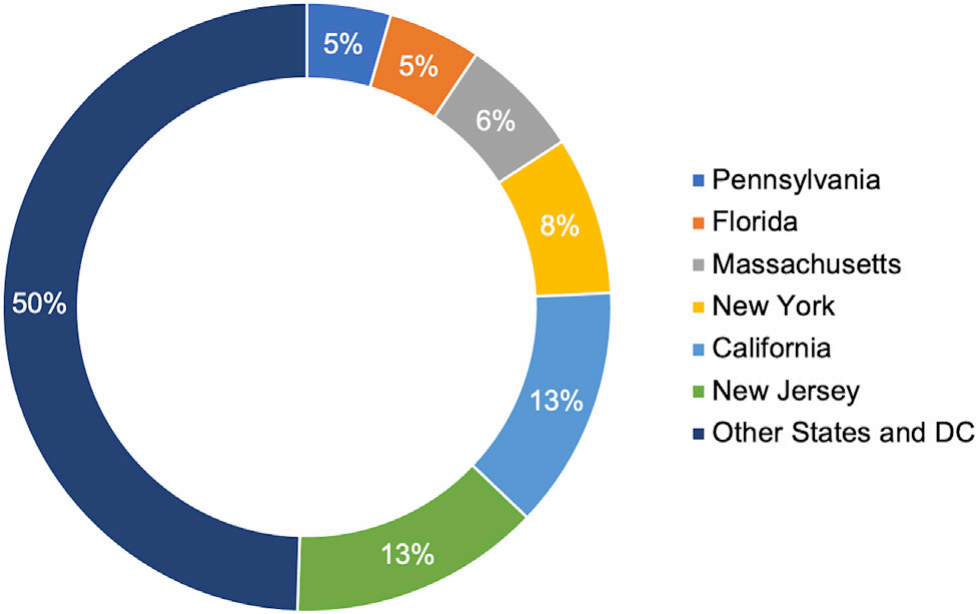
Jurisdictions in which genomic malpractice cases have been decided. Status is current through December 31, 2016.

**Figure 3:**
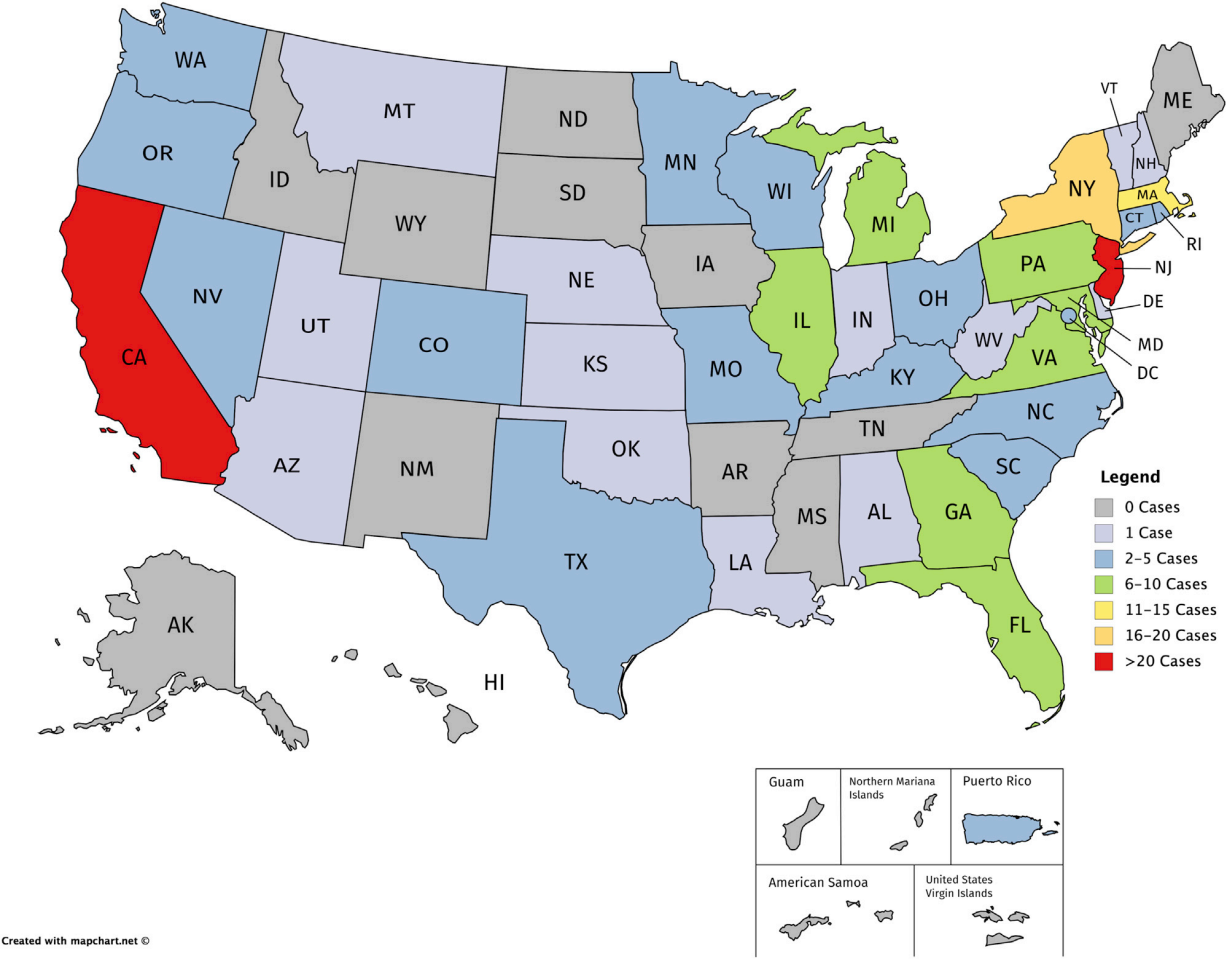
Number of genomic malpractice cases decided within each jurisdiction Status is current through December 31, 2016. Grey shading indicates zero cases. Lavender shading indicates one case. Blue shading indicates between two and five cases. Green shading indicates between six and 10 cases. Yellow shading indicates between 11 and 15 cases. Peach shading indicates between 16 and 20 cases. Red shading indicates more than 20 cases.

The previous empirical study of genomic malpractice cases (without regard to the LOC doctrine) conducted by Marchant and Lindor^1^ identified 202 cases through December 31, 2016. Of those, approximately 70% involved prenatal or newborn genetic testing, and 30% involved diagnostic, susceptibility, and pharmacogenomic testing. Reexamination of each case’s jurisdiction revealed that six states accounted for more than half (n = 102) of the genomic malpractice cases identified (California, Florida, Massachusetts, New Jersey, New York, and Pennsylvania), as shown in Figure 2. The LOC doctrine has been adopted in four of these jurisdictions (Massachusetts, New Jersey, New York, and Pennsylvania) and rejected in one of these jurisdictions (Florida). The number of cases decided within each jurisdiction is illustrated in Figure 3, with 29 of the 56 jurisdictions (states, district, and territories) having decided zero cases or only one case.

Of the disease conditions involved in these 202 genomic malpractice cases, three conditions accounted for one-third of the cases (Down syndrome [MIM: 190685], 36 cases; cystic fibrosis [MIM: 291700], 18 cases; and Tay-Sachs disease [MIM: 272800], 11 cases). Fourteen of the 202 genomic malpractice cases involved adult-onset conditions, including hereditary breast and ovarian cancer (HBOC) (MIM: 114480, MIM: 167000) involving *BRCA1* (MIM: 117305) or *BRCA2* (MIM: 600185); Lynch syndrome (MIM: 120435) involving *MLH1* (MIM: 120436), *MSH2* (MIM: 609309), *MSHG* (MIM: 600678), and *PMS2* (MIM: 600259); and *MUTYH*-associated polyposis (MIM: 604933). Of the 197 genomic malpractice cases for which source materials could be examined for this purpose (Table S2), the LOC doctrine was explicitly invoked in source materials for only five of the cases and possible, though not explicitly apparent, in source materials for an additional three cases. Thus, based on this analysis, the LOC doctrine was invoked in fewer than 5% of the genomic malpractice cases.

Four of the five cases in which it is clear that the LOC doctrine had been invoked involved allegations of a lost chance related to pregnancy termination.^37^, ^38^, ^39^, ^40^, ^41^ Of these four,^37^, ^38^, ^39^, ^40^ three involved claims of wrongful birth, in which the parents alleged they were deprived of the chance to avoid the financial and emotional costs of giving birth to a baby with a genetic condition.^37^, ^38^, ^39^, ^40^ One of these three cases^37^ involved an additional claim of wrongful life brought on behalf of the child. In the fourth case,^40^ the plaintiff terminated her pregnancy after 30 weeks’ gestation and was awarded damages for a lost chance to have an earlier abortion. The fifth case^41^ that clearly invoked the LOC doctrine involved allegations of a lost chance for parents of a child with Fanconi anemia (MIM: 227650) to give birth to another child who could serve as a sibling bone marrow transplant donor. Of the three additional cases in which the LOC doctrine is suggested, two involved a lost chance to terminate a pregnancy and the third involved allegations that delayed Turner syndrome (MIM: 300082) diagnosis caused a lost chance to avoid permanent short stature with human growth hormone therapies.

## Discussion

Scholars have for some time speculated that plaintiffs might rely on the LOC doctrine in genetic malpractice cases.^29^^,^^30^ To our knowledge, however, no empirical investigation until now has examined this question. This study has confirmed that there have indeed been attempts to rely upon the LOC doctrine by plaintiffs in genomic malpractice cases. This is a noteworthy finding, even if reliance upon the LOC doctrine is uncommon (as we observed) and even if legal scholars might disagree as to whether the facts merit invocation of the doctrine. Moreover, our methodological approach to determining how frequently genomic malpractice cases invoke the doctrine might have resulted in an underestimate: we did not review pleadings to initiate a lawsuit, which tend to encompass a broader set of claims than those that eventually make it to the jury verdicts, settlements, and court opinions we did review.

Defensive medicine—“when doctors order tests, procedures, or visits, or avoid certain high-risk patients or procedures, primarily (but not solely) because of concern about malpractice liability”^42^—reportedly varies considerably by medical specialty.^43^ The costs associated with defensive medicine have been estimated at 2.4% of total health care spending in the United States or approximately $45.59 billion in 2008 US dollars.^42^^,^^44^ Defensive medicine practices that one might anticipate in response to LOC doctrine-based liability risks include, for example, ordering more extensive genetic testing than is directly relevant for answering the clinical question(s) involved and data dumping (i.e., providing rapid access to raw genotype or genomic sequence data even if not interpreting or explaining those data or otherwise providing the data in a way that is readily understandable by patients without learned intermediaries). The rationale behind data dumping as a possible defensive practice, for example, is that if an individual has been given full access to any genetic/omic data (not merely given the interpretations for pathogenic conditions that are considered medically actionable at the time the result is obtained), the individual presumably has the ability to monitor the scientific literature personally or hire a professional to do so on the individual’s behalf, thereby (1) blunting the force of future allegations that genomic information was withheld and thereby caused the individual to lose chances to engage in preventative and risk-reducing behaviors and (2) potentially enabling future providers to raise contributory negligence as a defense (that is, to argue that the outcome was the result of the individual’s own doing).

Notably, it does not necessarily follow from the fact that a jurisdiction recognizes the LOC doctrine that a failure to recontact a patient with new or updated genomic information (such as when a variant of unknown significance is reclassified as pathogenic or likely pathogenic) opens the provider up to damages for any LOC the patient might have thereby suffered. Recall that a successful genomic medical malpractice claim requires the plaintiff to prove all elements of the cause of action: a duty existed, the defendant breached that duty, and the breach caused damages. A jurisdiction’s recognition that a lost chance resulting from a defendant’s breach of duty can constitute recoverable damages, satisfying the last element, is irrelevant unless the plaintiff can prove that a duty existed in the first place (the first element). Although some scholars argue that an ethical duty to reinterpret and/or recontact exists,^7^ US courts to date have recognized a duty of providers to recontact patients with new information in only narrow circumstances.^3^^,^^8^ Researchers are even less likely to be found to have a legal duty to recontact patient-participants,^16^ particularly in the absence of consistency or harmonization of policies governing researchers’ professional responsibilities regarding return of results.^6^

To mitigate LOC doctrine-related liability risks, genomic medicine providers or institutions might pursue other practices—not, strictly speaking, examples of defensive medicine—that likely would be in stark opposition to the growing sentiment favoring patient-centeredness (i.e., “providing care that is respectful of and responsive to individual patient preferences, needs, and values and ensuring that patient values guide all clinical decisions” (see Institute of Medicine^45^ at 6). These practices include increasing the length and complexity of consent to treat documents to incorporate additional disclosures, narrow the scope of services to be performed, and eliminate burdens of responsibility or shift such burdens onto patients through waivers of or releases from liability (i.e., contractually having patients explicitly assume risks). While informed consent documents *for care* often contain releases from liability (such as releases pertaining to potential health complications that are outside of the control of the medical professional performing a procedure or administering treatment), informed consent documents *for research participation* cannot include any exculpatory language (i.e., language that attempts to have research participants waive their legal rights or otherwise release researchers from tort liability).

While certainly not the only factor, the availability of the LOC medical malpractice doctrine is an important factor to consider when making programmatic decisions for genomic medicine. Currently, there are many opportunities for genomic risks to fall through the proverbial cracks. Increasing individual access to genomic data (regardless of the current scientific understanding as to how to interpret those data) could be a meaningful component of an effective risk mitigation strategy in both the health and legal liability sense, enabling individuals to monitor the ever-changing scientific evidence on their own (or through licensed experts acting on their behalf) and removing the informational disparity contributing to genomic professionals’ present precarious situation as to ongoing and uncertain risks of the LOC doctrine’s applicability to their patients’ health outcomes. Such a patient-centered approach, facilitated by the 21^st^ Century Cures Act and Blue Button 2.0 implementation,^46^^,^^47^ could usher in a suite of genome *monitoring* services not yet available and arguably too burdensome to be imposed directly upon genomic medicine providers at this time. Genomics spans several domains, including not only clinical medicine but also research, public health, and direct-to-consumer and other commercial domains, each governed by distinct legal frameworks.^9^ It is important to keep in mind that although courts have considered the LOC doctrine in areas beyond medical malpractice, including other forms of professional malpractice, torts, product liability, business, and employment, some jurisdictions might ultimately determine that the doctrine is unavailable in those other areas, even if it is permitted in medical malpractice.

### Conclusions

As genomic medicine and translational research mature, practitioners understandably worry about how medical malpractice law might pertain to their activities. The LOC doctrine has the potential for particularly broad application to genomic medicine, since (assuming all other elements are satisfied) it permits plaintiffs to recover based on professional acts and omissions, notwithstanding an underlying genetic condition with high penetrance that therefore strongly predisposed the plaintiff to injury regardless of the professional’s conduct. The LOC doctrine itself is evolving alongside genomic medicine. We observed a modest trend among US jurisdictions toward adopting the doctrine as part of their overall approach to medical malpractice (see Table S3), and we identified for the first time genomic malpractice cases in which the doctrine was invoked. Those five cases in which the doctrine was clearly invoked—of which four involved reproductive decisions—comprise fewer than 5% of all genomic malpractice cases identified through the end of 2016. As both genomic medicine and the LOC doctrine continue to evolve and expand, their intersection merits watching.

## Declaration of interests

Both J.K.W. and M.N.M. are employed by Geisinger, an integrated health care system where genomic medicine is practiced; however, they are research faculty and are not affiliated with the institution’s legal department or delivery of care. J.K.W. has a part-time private law practice in Pennsylvania, but this practice does not involve medical malpractice work. J.K.W. is also an academic affiliate of Anthropology at Penn State University. The authors declare no other competing interests.

## Data and code availability

The submitted manuscript includes all datasets generated or analyzed during this study.

## Web resources

mapchart, https://mapchart.net/
OMIM, https://omim.org/
WestlawNext, https://next.westlaw.com

## Supplemental information

Table S1. Relevant cases in determining the status of the loss of chance doctrine among the 50 states, District of Columbia, and five territories of the United States Displayed are the citations for the major cases contributing to the current categorization of the LOC doctrine in each jurisdiction

Table S2. Genomic malpractice cases included in the secondary analysis Displayed is the case name, citation, and research determination as to whether the LOC doctrine had been invoked

Table S3. Pinpoint citations for LOC doctrine status changes Displayed is more specific information (i.e., pinpoint citations) for each of the eight instances in which we reported a different status than Guest, Schap, and Tran
